# Baseline Composite Score for 12-Month Clinical Remission in Biologic-Treated Severe Asthma: Development of the Base4Score

**DOI:** 10.3390/biomedicines14040747

**Published:** 2026-03-25

**Authors:** Juan Luis García-Rivero, Adil Hannaoui Anaaoui, Abel Pallarés-Sanmartín, Marina Blanco-Aparicio, Raquel García-Hernáez, Victoria García-Gallardo Sanz, Uxío Calvo-Álvarez, Luis Carazo-Fernández, Tamara Hermida-Valverde, Silvia Dorronsoro, Inés Carrascosa-Anguiano, Ignacio Lobato Astiárraga, Idania de los Santos, Ana Isabel Enríquez Rodríguez, Luis Pérez de Llano, Pablo Álvarez Vega, Beatriz Abascal-Bolado, Miguel Santibañez

**Affiliations:** 1 Respiratory Department, Marqués de Valdecilla University Hospital, 39008 Santander, Spain; beatriz.abascal@scsalud.es; 2 Valdecilla Research Institute (IDIVAL), 39008 Santander, Spain; adil.hannaoui@idival.org; 3 Respiratory Department, Complejo Hospitalario Universitario de Vigo, 36312 Vigo, Spain; apallare@hotmail.com; 4 Respiratory Department, Hospital Universitario de A Coruña, 15006 A Coruña, Spain; mbamundo29@gmail.com; 5 Respiratory Department, Hospital Universitario San Pedro, 26006 Logroño, Spain; raquelmanjarres@hotmail.com; 6 Respiratory Department, Hospital Universitario de Burgos, 09006 Burgos, Spain; 7 Respiratory Department, Hospital Clínico Universitario de Santiago de Compostela, 15076 Santiago de Compostela, Spain; uxio.calvo.alvarez@sergas.es; 8 Respiratory Department, Hospital Universitario de León, 24008 León, Spain; luiscarazo@yahoo.es; 9 Respiratory Department, Hospital Universitario Central de Asturias, 33011 Oviedo, Spain; tamara.hermida.v@gmail.com (T.H.-V.); anaisenriquez@hotmail.com (A.I.E.R.); 10 Respiratory Department, Hospital Universitario de Donostia, 20014 Donostia/San Sebastián, Spain; silvia.dorronsoroquintana@osakidetza.eus; 11 Respiratory Department, Hospital de Urduliz, 48610 Urduliz, Spain; inescarrascosa03@gmail.com; 12 Respiratory Department, Hospital Río Carrión, 34005 Palencia, Spain; ignacio.lob.ast@gmail.com; 13 Respiratory Department, Hospital de Mendaro, 20850 Garaartza, Spain; idania7777@hotmail.com; 14 Respiratory Department, Hospital Lucus Augusti, 27003 Lugo, Spain; eremos26@hotmail.com; 15 Respiratory Department, Hospital Universitario de Cabueñes, 33394 Gijón, Spain; pabloalvega@gmail.com; 16 Global Health Research Group, Departamento Enfermeria, Faculty of Nursing, Universidad de Cantabria-IDIVAL, Avda. Valdecilla, s/n, 39008 Santander, Spain; miguel.santibanez@unican.es; 17 Centro de Investigación en Red de Enfermedades Respiratorias (CIBERES), Instituto de Salud Carlos III (ISCIII), 28029 Madrid, Spain

**Keywords:** severe asthma, biologics, remission, baseline severity, treat-to-target, prediction model

## Abstract

**Background:** Clinical remission has become a realistic treatment goal in severe asthma, but current evidence mostly reports global remission rates without accounting for baseline disease burden. No simple tool exists to quantify baseline severity and estimate an individual patient’s probability of achieving remission under biologic therapy. **Methods:** This prospective observational study included 93 adults with severe asthma initiating tezepelumab across 14 specialised severe asthma units in Spain. Four baseline domains—poor symptom control (ACT < 20), ≥1 severe exacerbation in the previous 12 months, maintenance oral corticosteroid (OCS) use, and FEV_1_ < 80% predicted—were used to construct an empirically weighted composite score (Base4Score) based on the inverse probability of correcting each abnormal domain at 12 months. Strict clinical remission at 12 months was defined as ACT ≥ 20, no severe exacerbations, no maintenance OCS, and FEV_1_ ≥ 80%. Logistic regression was used to assess the association between the score and non-remission, adjusting for age, sex, smoking status, T2 phenotype, and biologic-naive status. **Results:** Of 93 treated patients, 81 had complete baseline data for Base4Score derivation and 77 had complete 12-month data for strict clinical remission analysis. Strict clinical remission was achieved in 16/77 patients (20.8%). Remission rates decreased across increasing baseline score strata: 40.0% for scores < 5, 17.6% for scores 5 to <9, and 12.5% for scores ≥ 9 (linear *p*-trend = 0.022). Each 1-point increase in the continuous Base4Score was associated with higher adjusted odds of non-remission (OR 1.22; 95% CI 1.00–1.49; *p* = 0.047), and patients with scores ≥ 9 had approximately sevenfold higher adjusted odds of non-remission than those with scores < 5 (OR 6.77; 95% CI 1.40–32.84; *p* = 0.018). **Conclusions:** The Base4Score is a simple, empirically derived baseline severity index that predicts 12-month strict clinical remission in severe asthma treated with tezepelumab. If externally validated, it could help personalise expectations, optimise timing of biologic initiation and guide treat-to-target strategies in severe asthma.

## 1. Introduction

Asthma is a chronic inflammatory disease of the airways characterized by variable and reversible airflow obstruction, airway hyperresponsiveness, and respiratory symptoms triggered by a wide range of environmental and endogenous factors [1]. It represents a major global health problem, affecting hundreds of millions of individuals worldwide and accounting for a substantial burden in terms of healthcare utilization, work productivity loss, and reduced quality of life [2].

Severe asthma is a heterogeneous and disabling condition that remains uncontrolled in a substantial proportion of patients despite high-dose inhaled corticosteroids and additional controller therapies [3,4,5]. Over the past decade, biologic therapies targeting type 2 (T2) inflammation have transformed the management of severe asthma, leading to marked reductions in exacerbations, oral corticosteroid (OCS) use and symptom burden in many patients [6,7]. In this context, the concept of clinical remission—defined by sustained absence of exacerbations, steroid independence, good symptom control and preserved lung function—has emerged as a realistic and clinically meaningful treatment target rather than a purely aspirational goal [8,9,10].

International guidelines and expert consensus statements have progressively incorporated remission into their frameworks, proposing multidimensional definitions that typically include symptom scores (e.g., ACT or ACQ), severe exacerbations, maintenance OCS use and, in some cases, lung function thresholds [10,11,12]. These initiatives represent an important step towards a treat-to-target strategy in severe asthma. However, most clinical trials and real-world studies still report remission as a group-level outcome (for example, “X% of patients achieve clinical remission at 12 months with a given biologic”) without adequately accounting for the marked heterogeneity in baseline disease burden among treated patients [8,13,14,15].

Several analyses have identified individual baseline predictors associated with higher or lower probability of remission under biologic therapy. Lower baseline FEV_1_, more frequent exacerbations, OCS dependence, poorer symptom control, obesity and longer asthma duration have all been linked to reduced remission rates [16,17]. These observations suggest that where the patient starts may be as important as which biologic is used when interpreting remission. Yet, these variables are usually examined in isolation, and there is still no simple, pragmatic tool that integrates them into a single baseline severity score that can be easily applied in routine practice to personalise expectations and guide treatment decisions [10,11,18].

To address this gap, we designed a baseline composite score (Base4Score) built on four core clinical domains—symptom control, exacerbation history, maintenance OCS use, and lung function—that mirror the main components of current remission definitions and are readily available in daily practice. In the present study, we evaluate the 12-month performance of the Base4Score in a real-world cohort of patients with severe asthma treated with tezepelumab. By deriving empirical weights for each baseline domain based on its probability of correction at 12 months and examining the relationship between the resulting score and strict clinical remission, we aim to move beyond global remission rates and provide a simple, clinically grounded tool for baseline risk stratification.

The primary objective of this exploratory study was to develop an empirically weighted baseline composite score (Base4Score) to predict 12-month strict clinical remission in severe asthma patients receiving tezepelumab in routine practice. Secondary objectives were to characterise how individual baseline domains differentially contributed to the difficulty of achieving remission and to explore the potential of the score to inform remission probability and expectations at the individual patient level.

## 2. Methods

### 2.1. Study Design and Population

This was a multicentre, real-world analysis of prospectively collected data from a severe asthma registry including 93 adult patients with severe asthma who initiated tezepelumab in 14 specialised severe asthma units across Spain between 1 October 2023 and 1 October 2024. Severe asthma was defined according to GINA criteria, based on a documented diagnosis of asthma with variable airflow limitation, persistent symptoms and/or exacerbations despite high-dose inhaled corticosteroids plus at least one additional controller, and the need for or failure of maintenance oral corticosteroids.

Eligible patients were ≥18 years of age, had a confirmed diagnosis of severe asthma, initiated tezepelumab during the study period, and received treatment for a minimum duration of 12 months. Patients were required to have complete baseline data for the four domains needed to calculate the Base4Score—FEV_1_, ACT score, history of severe exacerbations in the previous 12 months, and maintenance oral corticosteroid (OCS) use.

The primary remission analysis was conducted in the subset of patients with complete 12-month follow-up data for all components of the strict clinical remission definition. Strict clinical remission at 12 months was defined as the simultaneous fulfilment of the following criteria: ACT ≥ 20, absence of severe exacerbations, no maintenance OCS, and FEV_1_ ≥ 80% predicted. Patients continued standard background asthma therapy, including inhaled corticosteroids and long-acting bronchodilators, in accordance with current clinical practice guidelines.

**Figure 1:**
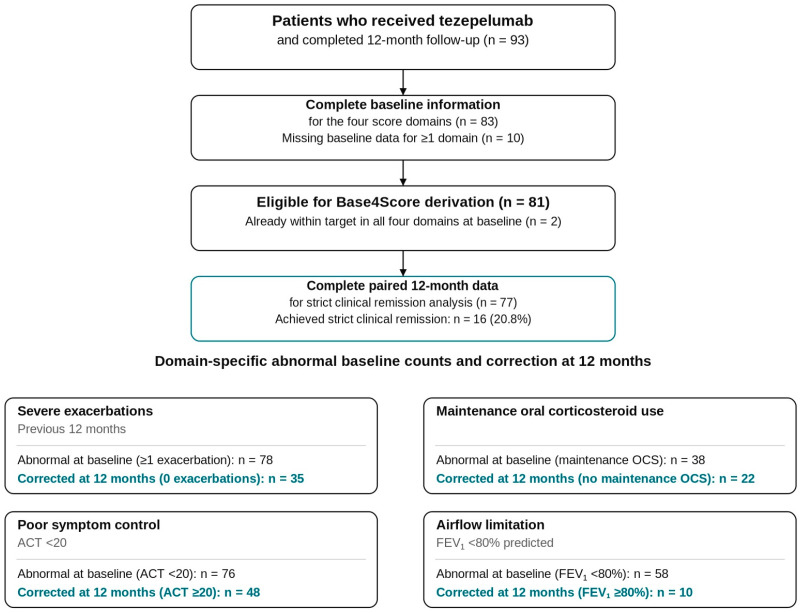
Flow diagram of patient inclusion and final analysis population for Base4Score derivation and 12-month strict clinical remission analysis.

Patient inclusion, exclusions, and derivation of the final analysis population are detailed in Figure 1. A total of 83 patients had complete baseline information for the four domains required to compute the Base4Score. Of these, 2 already met all remission criteria at baseline and therefore did not contribute to score derivation; the baseline population eligible for Base4Score derivation was thus 81 patients. At 12 months, 77 patients had complete paired data for all remission domains and constituted the primary analysis set for strict clinical remission. Baseline characteristics of patients finally included (*n* = 77 with valid paired data) and those excluded are compared in Supplementary Table S1.

Written informed consent was obtained from all participants prior to inclusion.

Tezepelumab was selected according to approved eligibility criteria and current clinical practice guidelines. The decision to initiate tezepelumab was made by the treating physician in routine clinical practice, based on individual patient characteristics, prior treatment history, and guideline-recommended indications, rather than for study-specific purposes. Type 2 inflammatory status was defined according to internationally accepted criteria and commonly used thresholds in severe asthma [1,5,19]. Patients were classified as T2-high if at least one of the following features was present at baseline: blood eosinophil count ≥ 150 cells/µL, fractional exhaled nitric oxide (FeNO) ≥ 25 ppb, and/or evidence of clinically relevant allergic sensitization. These biomarkers and thresholds are consistently endorsed by international guidelines and expert consensus documents as indicators of type 2 airway inflammation in severe asthma and are widely applied in clinical trials and real-world studies. Patients not meeting any of these criteria were classified as T2-low.

### 2.2. Construction of the Empirical Baseline Score (Base4Score)

Four baseline clinical domains were preselected a priori based on their central role in guideline-defined severe asthma, their consistent association with outcomes in previous remission studies, and their availability in routine practice:

1. Poor symptom control (ACT < 20);
2. ≥1 severe exacerbation in the previous 12 months;
3. Maintenance oral corticosteroid (OCS) use;
4. Airflow limitation (FEV_1_ < 80% predicted).

These domains mirror the core components included in current remission frameworks (symptoms, exacerbations, steroid independence and lung function) and have repeatedly been identified as baseline predictors of lower remission probability in recent clinical and real-world studies. We intentionally restricted the score to these four items to preserve simplicity and facilitate bedside use, accepting a trade-off between parsimony and completeness.

The Base4Score was designed to quantify baseline distance from strict clinical remission. Accordingly, domain weights were derived from the probability of correcting each abnormal baseline remission component at 12 months, rather than from the probability of remaining normal among patients already meeting that component at baseline. Domains already within the remission target at baseline contributed 0 points, because the score was conceived as a measure of baseline disease burden and reversibility needs rather than as a full transition model of all possible trajectories.

For each domain, we first dichotomised patients into “abnormal” (e.g., ACT < 20, ≥1 prior exacerbation, maintenance OCS, FEV_1_ < 80%) versus “normal” at baseline. We then calculated, among patients in the abnormal category at baseline, the proportion who achieved met the corresponding remission criterion at 12 months. This proportion represents the probability of successfully “reversing” that abnormal domain under tezepelumab treatment.

Empirical weights were derived using the inverse correction rate method: for each abnormal domain, we computed 1 ÷ (12-month correction probability). These values were then kept as decimal weights (rounded to one decimal place), so that domains that were harder to reverse (i.e., lower correction probability, such as FEV_1_ < 80%) received higher weights, reflecting a greater contribution to baseline disease burden, whereas domains that were easier to reverse (e.g., poor symptom control) received lower weights. The total Base4Score for each patient was defined as the sum of the weights for all abnormal baseline domains, with higher scores indicating greater baseline severity; in our sample, the score ranged approximately from 1.5 to 11.0 points.

For descriptive purposes, we also examined the probability of “remaining well” among patients in the normal category at baseline (e.g., ACT ≥ 20, FEV_1_ ≥ 80%), but these probabilities were not used to assign baseline severity weights. The final weights rounded to one decimal place and the underlying remission probabilities for each domain are presented in Table 1.

### 2.3. Statistical Analysis

The total baseline Base4Score ranged from 1.5 to 11.0 points in our sample. The primary analysis treated the Base4Score as a continuous predictor, and crude and adjusted odds ratios (ORs) with 95% confidence intervals (CIs) were obtained using logistic regression models to estimate the risk of not achieving and met the corresponding remission criterion at 12 months per 1-point increase in the score. Age (continuous), sex, smoking status (never, former, current), T2-high phenotype (yes/no) and biologic-naïve status (yes/no) were included in multivariable models as confounding variables.

For comparative purposes, the Base4Score was additionally normalised to a 1–4 scale using Min–Max transformation, so that its unit range matched that of a simple, unweighted score assigning 1 point to each abnormal domain (range 1–4). We then compared the performance of three predictors: (i) the original continuous Base4Score, (ii) the normalised Base4Score (1–4), (iii) the unweighted 0–4 score.

Finally, the Base4Score was categorised into three ordinal strata (<5, >4 to <9, and ≥9 points) to estimate stratum-specific ORs for non-remission, using the lowest category (<5) as the reference. A test for linear trend across categories was performed by including the ordinal score as a continuous term in the logistic model. A two-sided *p*-value < 0.05 was considered statistically significant. Analyses were performed using SPSS statistical software package 22.0 (SPSS, Inc., Chicago, IL, USA) and R, version 4.5.2 (R Foundation for Statistical Computing, Vienna, Austria), run via RStudio, version 2025.9.1.401 (Posit Software, PBC, Boston, MA, USA).

### 2.4. Ethics Considerations

The study was conducted in accordance with the Declaration of Helsinki and Spanish regulations for observational research. In Spain, multicentre non-interventional studies with medicinal products are reviewed and approved by a single reference Clinical Research Ethics Committee (CEIm), whose approval is legally binding for all participating centres, in accordance with Spanish Law 14/2007 on Biomedical Research and Royal Decree 957/2020. Accordingly, the study was reviewed and approved by the Clinical Research Ethics Committee of Cantabria (CEIm Cantabria), which acted as the reference ethics committee (protocol code 2024.090, approval date: 12 April 2024). All participating centres were formally informed and agreed to take part in the study under the same approved protocol.

## 3. Results

### 3.1. Baseline Characteristics

Baseline characteristics of the 81 patients included in the score analysis and the final 77 patients are summarised in Table 2. In brief, this was a typical real-life severe asthma cohort: patients were predominantly middle-aged and female, with a high prevalence of obesity and type 2 comorbidities such as allergic rhinitis, chronic rhinosinusitis with nasal polyps and bronchiectasis. Most patients were receiving high-dose ICS/LABA and nearly half were on maintenance OCS at baseline. Both T2-high and T2-low phenotypes were represented, reflecting the heterogeneity of severe asthma in routine practice.

### 3.2. Domain-Specific Remission Probabilities and Empirical Weights

At baseline, most patients presented abnormalities in several clinical domains. Among the 81 patients with complete data, over 90% had either ≥1 severe exacerbation in the previous 12 months and/or ACT < 20, approximately 64%% had FEV_1_ < 80% predicted, and about 41% were on maintenance OCS.

When each domain was analysed separately, the probability of “improvement” achieving a correction at 12 months differed substantially. The following are seen in Figure 1 and Table 1:

- Among patients with ≥1 severe exacerbation in the prior year, 35/76 (46.1%) had no severe exacerbations during follow-up.
- Among those on maintenance OCS, 22/36 (61.1%) were able to discontinue OCS by 12 months.
- Among patients with poor symptom control (ACT < 20) at baseline, 48/73 (65.8%) achieved ACT ≥ 20.
- In contrast, only 10/57 (17.5%) of those with FEV_1_ < 80% predicted at baseline reached FEV_1_ ≥ 80% at 12 months.

Using the inverse of these remission probabilities, we derived empirical weights that quantify the “difficulty” of reversing each abnormal baseline domain. The resulting weights were 5.7 points for FEV_1_ < 80%, 2.2 for ≥1 prior exacerbation, 1.6 for maintenance OCS use and 1.5 for ACT < 20, yielding a continuous score ranging approximately from 1.5 to 11 points (Table 1). Domains that were more resistant to change, particularly impaired lung function, contributed disproportionately to the overall baseline severity score.

### 3.3. Distribution of the Base4Score

Supplementary Table S2 shows the cumulated number of domains susceptible of improvement, this is, the unweighted 1–4 score, in the final study population with paired valid data (*n* = 77). The distribution of the continuous Base4Score in the 81 and 77 patients is shown in Supplementary Table S3. Scores spanned the full range, with clustering around medium–high values, reflecting the high baseline burden of disease in this cohort. For clinical interpretation, we predefined three strata of baseline severity:

- Low severity: score <5 (20/77, 26.0%).
- Intermediate severity: 5 to <9 (17/77, 22.1%).
- High severity: ≥9 (40/77, 51.9%).

These categories captured a clinically meaningful gradient of baseline disease burden and were used for subsequent risk-stratified analyses.

### 3.4. Association Between Baseline Score and 12-Month Strict Clinical Remission in the 4 Domains

**Figure 2:**
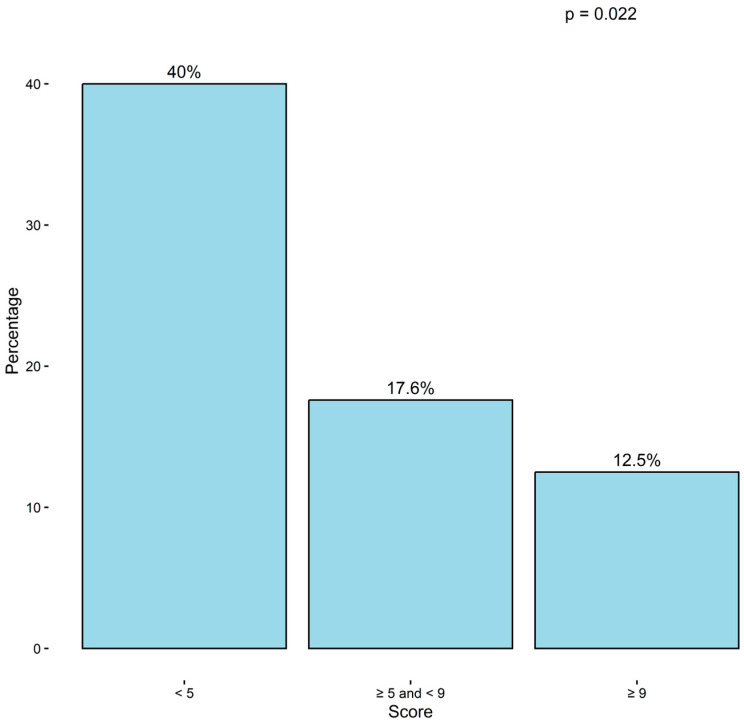
**Twelve-month strict clinical remission rates according to baseline Base4Score strata.** Strict clinical remission was achieved in 40.0% of patients with low baseline scores (<5), 17.6% with intermediate scores (5 to <9), and 12.5% with high baseline scores (≥9). A significant trend across categories was observed (linear *p*-trend = 0.022).

Strict clinical remission at 12 months (ACT ≥ 20, no severe exacerbations, no maintenance OCS, and FEV_1_ ≥ 80% predicted) was achieved in 16 of 77 patients (20.8%) with complete paired outcome data. When analysed by baseline severity strata, the probability of achieving strict clinical remission decreased sharply with increasing Base4Score (Figure 2). Remission occurred in 8/20 (40.0%) of patients with low baseline scores (<5), 3/17 (17.6%) of those with intermediate scores (5–<9), and only 5/40 (12.5%) of patients with high scores (≥9), with a significant trend across categories (linear *p*-trend = 0.022).

In logistic regression, each 1-point increase in the continuous Base4Score was associated with 1.22 higher adjusted odds of strict clinical remission (OR 1.22; 95% CI 1.00–1.49; *p* = 0.047). When the score was standardised to a 1–4 scale, each unit increase approximately doubled the risk of non-remission (ORa 1.91; 95% CI 1.01–3.63; *p* = 0.047). In contrast, the unweighted 1–4 score—assigning one point per abnormal domain without empirical weighting— showed a weaker association and did not reach statistical significance (Table 3).

Categorisation into the three predefined strata showed a clear risk gradient: patients with scores ≥9 had approximately sevenfold higher adjusted odds of non-remission than those with scores <5 (OR 6.77, 95% CI 1.40–32.84; *p* = 0.018), with a significant linear trend across categories (*p*-trend = 0.021). These findings confirm that the empirically weighted Base4Score captures clinically relevant differences in baseline severity that translate into markedly different probabilities of achieving strict clinical remission under biologic therapy (Table 4).

Baseline clinical severity, lung function, oral corticosteroid use, and key inflammatory biomarkers across Base4Score strata are summarised in Supplementary Table S4. This stratified analysis illustrates a clear gradient of clinical burden and biological profile with increasing baseline score, providing clinical context for the marked differences in remission probability observed across strata.

### 3.5. Exploratory Analyses of Inflammatory Remission

Exploratory analyses were performed for inflammatory remission using FeNO and blood eosinophils. Although baseline data were available for most patients, follow-up measurements at 12 months were missing in a substantial proportion (only 49 patients with complete FeNO and eosinophil data), limiting the robustness of these analyses. In this restricted subgroup, patterns were broadly consistent with the clinical score: patients with lower baseline Base4Score tended to show higher rates of combined clinical and biological remission, but numbers were too small to draw firm conclusions.

## 4. Discussion

In this multicentre real-world cohort of adults with severe asthma initiating tezepelumab, we developed an empirically weighted baseline severity score (Base4Score) and showed a clear, graded association with the likelihood of achieving strict clinical remission at 12 months. Using a stringent remission definition aligned with current multidimensional frameworks (ACT ≥ 20, no severe exacerbations, no maintenance OCS, and FEV_1_ ≥ 80% predicted) (10–12), strict clinical remission was achieved by 20.8% of patients. Importantly, remission rates differed markedly according to baseline burden: 40.0% in patients with low Base4Score (<5), 17.6% in the intermediate stratum, and 12.5% in the high stratum (≥9), supporting the concept that remission should be interpreted through a baseline-anchored lens rather than as a drug-only property.

Beyond stratified proportions, the Base4Score showed a clear dose–response association with non-remission in regression analyses (adjusted OR 1.22 per 1-point increase). However, because Base4Score units reflect empirically weighted domain reversibility, a 1-point increment does not correspond to a single, intuitive clinical change. For this reason, we emphasised interpretation using prespecified risk strata (<5, 5 to <9, and ≥9 points). Using this stratified approach, patients in the highest category had approximately seven-fold higher adjusted odds of non- remission compared with those in the lowest category. The empirically weighted score outperformed the simple unweighted 1–4 domain count, suggesting that accounting for differential domain reversibility adds information beyond the number of abnormal domains.

These findings complement and extend the growing literature on clinical remission under biologic therapy. Recent systematic reviews and observational analyses indicate that remission is achievable in a subset of biologic-treated patients but is heterogeneously defined and often reported as an overall group-level proportion [10,18]. Studies exploring predictors of remission consistently identify poorer baseline lung function, maintenance OCS use, and frequent exacerbations as markers of lower remission probability [16,18], and emerging tezepelumab real-world evidence supports the feasibility of combined clinical and biological improvement [13]. This is also aligned with our recent 12-month multicenter real-world experience with tezepelumab, in which strict clinical remission was more frequently achieved in patients with higher baseline T2 biomarker burden, particularly those with FeNO ≥ 25 ppb and/or blood eosinophils ≥ 150 cells/µL [20]. Importantly, clinically relevant benefit was still observed across phenotypes, reinforcing the need to interpret remission probability in light of both inflammatory profile and baseline disease burden. Our results align with these observations while providing a pragmatic way to integrate four routinely collected, remission-aligned domains into a single baseline severity index that allows bedside risk stratification.

A key insight from our empirical weighting approach is the differential correction of remission domains over 12 months. In our cohort, improvement in symptom control and discontinuation of maintenance OCS were relatively frequent, whereas normalisation of lung function to FEV_1_ ≥ 80% predicted was uncommon among those starting below that threshold. This translated into a substantially larger weight for the FEV_1_ domain, highlighting lung function as the dominant driver of baseline remission difficulty. Clinically, this pattern is consistent with the notion that chronic airflow limitation may reflect accumulated airway remodelling or fixed obstruction that is less amenable to reversal than exacerbation risk or symptom burden, even under effective biologic therapy. The score therefore operationalises a common clinical intuition: patients “starting further from the target,” particularly in lung function, are less likely to reach a strict multidimensional remission state at one year.

The clinical and laboratory characterisation of patients across Base4Score strata (Supplementary Table S4) further supports that increasing baseline scores reflect a cumulative burden of airflow limitation, exacerbation history, and treatment intensity, rather than an artefact of score construction.

From a practical perspective, the Base4Score may support more individualised counselling by translating baseline disease burden into different remission probabilities under the same biologic. This could help clinicians and patients contextualise treatment goals and interpret the absence of strict clinical remission in those with high baseline severity. Although the present study was not designed to establish causal effects of earlier initiation, the prominent contribution of impaired lung function to remission difficulty supports the hypothesis that initiating effective therapy before long-standing functional impairment becomes established may increase the chance of meeting stringent remission definitions. This hypothesis should be tested in larger cohorts with richer longitudinal data (including asthma duration and trajectories) and should not be interpreted as a treatment timing recommendation derived directly from our data.

Several asthma severity and burden-oriented indices have been proposed across different clinical contexts, reflecting the multidimensional nature of the disease. Acute severity scores, such as the Buddhasothorn Asthma Severity Score (BASS) and similar paediatric triage instruments, were developed to stratify the intensity of acute exacerbations and guide emergency department management [21]. These tools rely primarily on cross-sectional clinical signs to classify the severity of the current episode and support short-term treatment decisions. At the other end of the spectrum, Zein and colleagues recently introduced a longitudinal asthma health-care burden score derived from two large adult cohorts (SARP III and U-BIOPRED), integrating exacerbations, health-care utilisation, and rescue SABA use into a DALY-weighted composite index [22]. This dynamic score was designed to challenge pharmacologically based definitions of severe asthma and demonstrated strong performance in predicting clinical remission over time by capturing accumulated morbidity across longitudinal follow-up.

In parallel, the Asthma Severity Scoring System (ASSESS), validated in the ALLIANCE cohort across paediatric and adult populations, represents another multidimensional approach to asthma severity quantification [23]. ASSESS integrates symptom burden (ACT), lung function, medication intensity, and exacerbations into a weighted composite scale ranging from 0 to 20, showing good construct validity and responsiveness across age groups. Unlike acute triage tools, ASSESS was developed as a global severity instrument applicable across heterogeneous asthma populations, capturing multiple dimensions of disease expression within a continuous framework.

The Base4Score differs from these instruments in both purpose and construction. It is not intended as a triage tool for acute exacerbations, nor as a longitudinal health-care burden tracker, nor as a comprehensive severity index spanning the full asthma spectrum. Instead, it is a baseline stratification instrument applied at the moment of biologic initiation in stable severe asthma. It focuses exclusively on four remission-aligned clinical domains—symptom control, exacerbations, maintenance OCS use, and lung function—and assigns empirical weights derived from observed domain-specific reversibility under biologic therapy. Whereas health-care burden scores capture accumulated morbidity over time and ASSESS integrates multidimensional severity across varying treatment intensities, the Base4Score captures accumulated physiological and clinical impairment at baseline and translates it into differential probabilities of achieving a stringent multidimensional remission state at 12 months.

Thus, rather than competing with existing burden or severity indices, the Base4Score should be viewed as a complementary, remission-anchored framework tailored to treat-to-target strategies in severe asthma. Its conceptual simplicity and immediate bedside applicability distinguish it from more complex longitudinal or multidimensional models, while positioning it within the broader effort to refine how asthma severity and remission are defined and operationalised in clinical practice. Although the Base4Score was intentionally restricted to four remission-aligned clinical domains to preserve simplicity and bedside applicability, other factors such as obesity, age at asthma onset, asthma duration, and broader comorbidity burden may also influence the probability of remission and were not explicitly incorporated into the score.

Our study has limitations. First, this was a single-cohort analysis and performance was assessed internally only; external validation in independent populations and across other biologics is essential before clinical implementation. Second, the sample size was modest, and complete paired 12-month data were available in only 77 patients, which limited precision, widened confidence intervals, and restricted the feasibility of extensive subgroup analyses. Third, the strict clinical remission definition used in this study was intentionally demanding and may underestimate clinically meaningful benefit in patients who experience major improvements in exacerbations, symptoms, or OCS burden but remain below the FEV_1_ threshold [10,11,12]. Fourth, the use of an FEV_1_% predicted threshold (80%) is widely accepted but may imperfectly capture functional normalization across ages and baseline obstruction severity. Future studies should also evaluate the performance of the Base4Score using less stringent remission definitions, including clinically oriented definitions that do not require lung function normalisation, to determine how the score behaves across different remission frameworks. Finally, we did not incorporate biomarkers into the score; follow-up inflammatory data were missing in a substantial proportion, and any combined clinical-biological remission signal should be considered exploratory. In addition, because tezepelumab was prescribed according to the approved indication and the Spanish clinical positioning framework during the study period, the cohort may have been enriched for patients with substantial baseline disease burden, which should be considered when interpreting generalisability.

## 5. Conclusions

The Base4Score is a simple, empirically derived baseline severity index that predicts 12-month strict clinical remission in patients with severe asthma treated with tezepelumab. By weighting four routine clinical domains—lung function, symptom control, prior exacerbations, and maintenance OCS use—according to their probability of correction, the score captures clinically meaningful differences in baseline disease burden and translates them into markedly different remission probabilities. Our findings suggest that remission rates with biologics cannot be interpreted independently of where patients start and support a shift towards remission-oriented, baseline-anchored decision making. External validation in independent cohorts and with other biologics will be essential to confirm the generalisability of the Base4Score.

## Figures and Tables

**Table 1:** Domain-specific remission probabilities and empirical weights.

Baseline Domain	Abnormal at Baseline (%)	12-Month Improvement (%)	1/*p*	Weight
FEV_1_ < 80%	58/92 (63.0%)	10/57 (17.5%)	5.7	5.7
ACT < 20	76/85 (89.4%)	48/73 (65.8%)	1.5	1.5
≥1 exacerbation	78/92 (84.8%)	35/76 (46.1%)	2.2	2.2
Maintenance OCS	38/93 (40.9%)	22/36 (61.1%)	1.6	1.6

Denominators for “abnormal at baseline” and “12-month improvement” were calculated separately for each domain because baseline and paired 12-month data availability differed across variables. Therefore, the proportions shown are domain-specific rather than based on a single common denominator. The total Base4Score is the sum of the weights for abnormal baseline domains (range approximately 1.5 to 11.0). Weights were calculated using full precision and are displayed rounded to one decimal place for readability and clinical interpretation. Abbreviations: FEV_1_, forced expiratory volume in 1 s; ACT, Asthma Control Test; OCS, oral corticosteroids.

**Table 2:** Baseline characteristics of the 81 patients included in the score analysis and the final 77 patients.

Variable	Total (*n* = 93)	Basal Included * (*n* = 81)	12-Month Valid Paired Data (*n* = 77)
Age (y)	55.88 ± 14.95	57.05 ± 14.40	57.09 ± 14.57
Female	66 (70.97%)	56 (66.67%)	51 (66.23%)
BMI (kg/m^2^)	30.42 ± 6.45	30.33 ± 6.18	30.31 ± 6.28
*Smoking: never* (%)	58 (62.37)	49 (60.49%)	47 (61.04%)
*Phenotypic characteristics*			
* T2-low biomarkers*	31 (33.33%)	28 (34.57%)	26 (33.77%)
* Naïve T2-low biomarkers*	25 (26.88%)	22 (78.57%)	21 (80.77%)
* Biologic-switch T2-low biomarkers*	6 (6.45%)	6 (21.43%)	5 (19.23%)
* T2-high*	62 (66.67%)	53 (65.43%)	51 (66.23%)
* Naïve T2-high*	26 (41.94%)	21(39.62%)	19 (37.25%)
* Biologic-switch T2-high*	36 (58.06%)	32 (60.38%)	32 (62.75%)
* Allergic*	31 (33.33%)	27 (50.94%)	25 (32.47%)
* Eosinophilic*	31 (33.33%)	26 (49.06%)	26 (33.77%)
*Adult-onset*	65 (69.89%)	57 (70.37%)	55 (71.43%)
*Comorbidities*			
Obesity	50 (53.76%)	44 (54.32%)	42 (54.55%)
* Bronchiectasis*	21 (22.58%)	20 (24.69%)	18 (23.38%)
* COPD*	11 (11.83%)	10 (12.35%)	9 (11.69%)
*Medication*			
Patients with long-term OCS therapy	38 (40.86%)	33 (40.74%)	30 (38.96%)
ICS low	4 (4.3%)	2 (2.47%)	2 (2.6%)
ICS medium	9 (9.68%)	9 (11.11%)	9 (11.69%)
ICS high	79 (84.95%)	69 (85.19%)	66 (85.71%)
LABA	92 (98.92%)	80 (98.77%)	77 (100%)
LAMA	88 (94.62%)	76 (93.83%)	73 (94.81%)
Montelukast	46 (49.46%)	40 (49.38%)	39 (50.56%)
Macrolides	35 (37.63%)	35 (43.21%)	33 (42.86%)
Previous biologic treatments			
Biologic-naïve	51 (54.84%)	43 (53.09%)	40 (51.95%)
Previous Omalizumab	9 (9.68%)	8 (9.88%)	8 (10.39%)
Previous Mepolizumab	9 (9.68%)	9 (11.11%)	9 (11.69%)
Previous Benralizumab	10 (9.30%)	9 (11.11%)	7 (9.09%)
Previous Dupilumab	13 (13.98%)	13 (16.05%)	12 (15.58%)
Previous Tezepelumab	1 (1.08%)	1 (1.23%)	1 (1.30%)

Abbreviations: BMI, body mass index; SD, standard deviation; T2, type 2 inflammation; COPD, chronic obstructive pulmonary disease; OCS, oral corticosteroids; ICS, inhaled corticosteroids; LABA, long-acting β_2_-agonist; LAMA, long-acting muscarinic antagonist. * With complete baseline information for the four domains required to compute the Base4Score, and without clinical remission, this is, susceptible of improvement (correction) in at least one domain. Percentages are calculated using the column total unless otherwise specified. For naïve/biologic-switch T2-low rows, percentages are calculated within the T2-low subgroup; for naïve/biologic-switch T2-high and allergic/eosinophilic rows, percentages are calculated within the T2-high subgroup.

**Table 3:** Crude and adjusted odds ratios (ORs) for failure to achieve strict clinical remission according to each composite score treated as a continuous variable.

	ORc	95%	CI	Linear *p* Trend	ORa	95%	CI	Linear *p*-Trend
**Base4Score(range 1.5–11.0)**	1.21	1.01	1.45	0.043	1.22	1.00	1.49	0.047
**Base4Score standardised to 1–4**	1.83	1.02	3.29	0.043	1.91	1.01	3.63	0.047
**Unweighted 1–4 score**	1.68	0.89	3.16	0.11	1.88	0.95	3.72	0.068

ORc = Crude Odds Ratio. ORa = Odds Ratio adjusted for age (continuous), sex, smoking status (never/former/current), T2-high phenotype (yes/no) and biologic-naïve status (yes/no).

**Table 4:** Crude and adjusted odds ratios (ORs) for failure to achieve strict clinical remission according to ordinal categories of the Base4Score.

	ORc	95%	CI	*p* Value	ORa	95%	CI	*p* Value
**Base4Score (range 1.5–11.0 points)**								
**<5**	1				1			
**>4 to <9**	3.11	0.67	14.44	0.147	4.67	0.57	38.20	0.151
**≥9**	4.67	1.28	17.05	0.02	6.77	1.40	32.84	0.018
**Linear *p* trend**	0.022				0.021			

ORc = Crude Odds Ratio. ORa = Odds Ratio adjusted for age (continuous), sex, smoking status (never/former/current), T2-high phenotype (yes/no) and biologic-naïve status (yes/no).

## Data Availability

The data underlying this article are available from the corresponding author upon reasonable request. Raw data cannot be publicly shared due to patient privacy restrictions.

## Supplementary Materials

The following supporting information can be downloaded at: https://www.mdpi.com/article/10.3390/biomedicines14040747/s1, Table S1. Baseline characteristics of patients included vs. excluded from the strict clinical remission analysis; Table S2. Cumulated number of domains susceptible of improvement in the final study population with paired valid data (*n* = 77); Table S3. Distribution of the continuous Base4Score in the study population; Table S4. Baseline clinical and laboratory characteristics in the final paired population according to Base4Score strata.

## Abbreviations

ACT: Asthma Control Test; ACQ: Asthma Control Questionnaire; BMI: Body Mass Index; CRSwNP: Chronic Rhinosinusitis with Nasal Polyps; FEV1: Forced Expiratory Volume in 1 Second; GINA: Global Initiative for Asthma; GEMA: Guía Española para el Manejo del Asma; ICS: Inhaled Corticosteroids; LABA: Long-Acting Beta-Agonists; LAMA: Long-Acting Muscarinic Antagonists; OCS: Oral Corticosteroids; T2: Type 2 Inflammation.
