# Supplementary material for: Baseline Composite Score for 12-Month Clinical Remission in Biologic-Treated Severe Asthma: Development of the Base4Score
Source: Biomedicines. 2026 Mar 25;14(4):747. doi: 10.3390/biomedicines14040747 (PMC13113559; doi:10.3390/biomedicines14040747)
Supplement: Supplementary file 1 [file biomedicines-14-00747-s001.zip › biomedicines-4177845-SI.pdf]

Supplementary Table S1. Baseline characteristics of patients included vs. excluded from the strict clinical remission analysis.

Variable	Patients included (n=77)	Patients excluded (n=16)
Age (y) (mean ± SD)	57.09 ± 14.57	50.06 ± 15.85
Female (%)	51 (66.23%)	15 (93.75%)
BMI (kg/m^2) (mean ± SD)	30.31 ± 6.28	30.97 ± 7.42
Smoking: never (%)	47 (61.04%)	11 (68.75%)
T2-low (%)	26 (33.77%)	5 (31.25%)
T2-high (%)	51 (66.23%)	11 (68.75%)
Allergic (%)	25 (32.47%)	6 (37.5%)
Eosinophilic (%)	26 (33.77%)	5 (31.25%)
Obesity (%)	42 (54.55%)	8 (50%)
Bronchiectasis (%)	18 (23.38%)	3 (18.75%)
COPD (%)	9 (11.69%)	2 (12.5%)
Patients with long-term OCS therapy (%)	30 (38.96%)	8 (50%)
Biologic-naïve (%)	40 (51.95%)	11 (68.75%)
Baseline All severe Exacerbations (mean ± sd)	3.65 ± 2.43 (n = 77)	3.27 ± 2.6 (n = 15)
Baseline FEV1 pre-BD (mL) (mean ± sd)	1844.68 ± 777.6 (n = 77)	2110.67 ± 1018.91 (n = 15)
Baseline Eosinophils (n/mL) (mean ± sd)	232.79 ± 333.44 (n = 75)	102.53 ± 115.54 (n = 15)
Baseline IgE total (IU/mL) (mean ± sd)	162.75 ± 248.97 (n = 70)	141.26 ± 193.07 (n = 11)
Baseline FeNO (ppb) (mean ± sd)	23.08 ± 22.19 (n = 76)	30.67 ± 29.06 (n = 15)

Values are expressed as mean ± SD or n (%). The two groups were broadly comparable across baseline characteristics, suggesting that excluding was unlikely to introduce major selection bias.

Supplementary Table S2. Cumulated number of domains susceptible of improvement in the final study population with paired valid data (n=77)

Cumulated number of altered domains (unweighted 1-4 score)	n=77	%
1	6	7,8
2	20	26,0
3	33	42,9
4	18	23,4

Supplementary Table S3. Distribution of the continuous Base4Score in the study population

Base4Score (1.5-11.2)	n=81	%			n=77	%
1.5	3	3.7	3	3.8	3	3.9
2.4	1	1.2	1	1.3	1	1.3
3.9	16	19.8	16	20.3	16	20.8
4	1	1.2				
5.5	8	9.9	7	8.9	7	9.1
5.7	2	2.5	2	2.5	2	2.6
7.2	2	2.5	2	2.5	2	2.6
8.1	2	2.5	2	2.5	2	2.6
8.9	4	4.9	4	5.1	4	5.2
9.6	22	27.2	21	26.6	21	27.3
9.7	1	1.2	1	1.3	1	1.3
11.2	19	23.5	18	22.8	18	23.4

Supplementary Table S4. Baseline clinical and laboratory characteristics in the final paired population according to Base4Score strata.

Variable	12-month valid paired data (n = 77)	Base4Score < 5 (n = 20)	Base4Score 5 to <9 (n = 17)	Base4Score ≥ 9 (n = 40)
Age (y)	57.09 ± 14.57	56.1 ± 16.7	57.12 ± 13.03	57.58 ± 14.4
Female	51 (66.23%)	16 (80%)	7 (41.18%)	28 (70%)
BMI (kg/m²)	30.31 ± 6.28	29.45 ± 6.45	31.62 ± 6.78	30.16 ± 6.05
Smoking: never (%)	47 (61.04%)	14 (70%)	10 (58.82%)	23 (57.5%)
Phenotypic characteristics				
T2-low biomarkers	26 (33.77%)	11 (55%)	1 (5.88%)	14 (35%)
Naïve T2-low biomarkers	21 (80.77%)	9 (45%)	1 (5.88%)	11 (27.5%)
Biologic-switch T2-low biomarkers	5 (19.23%)	2 (10%)	0 (0%)	3 (7.5%)
T2-high	51 (66.23%)	9 (45%)	16 (94.12%)	26 (65%)
Naïve T2-high	19 (37.25%)	8 (88.89%)	1 (6.25%)	10 (38.46%)
Biologic-switch T2-high	32 (62.75%)	1 (11.11%)	15 (93.75%)	16 (61.54%)
Allergic	25 (32.47%)	4 (20%)	7 (41.18%)	14 (35%)
Eosinophilic	26 (33.77%)	5 (25%)	9 (52.94%)	12 (30%)
Adult-onset	55 (71.43%)	13 (65%)	13 (76.47%)	29 (72.5%)
Comorbidities				
Obesity	42 (54.55%)	9 (45%)	12 (70.59%)	21 (52.5%)
Bronchiectasis	18 (23.38%)	6 (30%)	3 (17.65%)	9 (22.5%)
COPD	9 (11.69%)	0 (0%)	4 (23.53%)	5 (12.5%)
Medication				
Patients with long-term OCS therapy	30 (38.96%)	0 (0%)	11 (64.71%)	19 (47.5%)

Patients were stratified into three predefined Base4Score categories (<5, 5 to <9, and ≥9 points). Data are presented as n (%) or mean ± SD, as appropriate. The table highlights gradients in baseline clinical severity, inflammatory phenotype, comorbidities, and treatment intensity across score strata.
